# Ovarian Innervation Coupling With Vascularity: The Role of Electro-Acupuncture in Follicular Maturation in a Rat Model of Polycystic Ovary Syndrome

**DOI:** 10.3389/fphys.2020.00474

**Published:** 2020-05-29

**Authors:** Xiaoyu Tong, Yanjun Liu, Xiaoqing Xu, Jiemei Shi, Wei Hu, Tong Ma, Peng Cui, Wenhan Lu, Zhenle Pei, Mingzhen Xu, Feifei Zhang, Xin Li, Yi Feng

**Affiliations:** ^1^Department of Integrative Medicine and Neurobiology, School of Basic Medical Sciences, Institutes of Brain Science, Brain Science Collaborative Innovation Center, State Key Laboratory of Medical Neurobiology, Fudan Institutes of Integrative Medicine, Fudan University, Shanghai, China; ^2^Department of Obstetrics and Gynecology, Shanghai Medical School, Fudan University, Shanghai, China; ^3^Shanghai Key Laboratory of Female Reproductive Endocrine Related Diseases, Fudan University, Shanghai, China; ^4^Department of Obstetrics and Gynecology, Shuguang Hospital Affiliated to Shanghai University of Traditional Chinese Medicine, Shanghai, China

**Keywords:** electro-acupuncture, ovarian innervation, follicular maturation, neurovascular coupling, CUBIC three-dimensional tissue clearing

## Abstract

Low-frequency electro-acupuncture (EA) has been shown to restore ovulation in patients with polycystic ovary syndrome (PCOS), and previous animal experiments showed that EA improves ovarian blood flow and angiogenesis. We performed EA for 4 weeks in dihydrotestosterone (DHT)-induced PCOS-like rats and investigated the three-dimensional (3D) ovarian innervation to determine the role of innervation in folliculogenesis and vascularity. Ovarian tissues were made transparent following the CUBIC 3D tissue-clearing protocol and were immunostained using antibodies against platelet endothelial cell adhesion molecule-1 and tyrosine hydroxylase to visualize the ovarian vasculature and innervation, respectively. This was followed by 3D imaging using lightsheet microscopy and analysis using the Imaris software. In control rats, ovarian innervation increased with age, and the neuronal branching started from the ovarian hilum and reached the individual follicles at different follicle stages. At the individual follicle level, each follicle was mainly innervated by one neuronal fiber. Compared with control rats, ovaries from DHT-treated PCOS-like rats had more antral follicles and fewer preovulatory follicles and corpora lutea. Furthermore, PCOS ovaries showed decreased innervation of blood vessels near the hilum and the surrounding individual antral follicles. EA in PCOS-like rats led to increased numbers of preovulatory follicles and corpora lutea together with increased innervation of blood vessels near the hilum. To determine the role of ovarian innervation, we further performed unilateral sectioning of the superior ovarian nerve (SON) in PCOS + EA rats and found that the left sectioned ovary had fewer preovulatory follicles and corpora lutea compared with those in the right non-sectioned ovary. In conclusion, ovarian innervation likely played an important role in folliculogenesis, and EA might restore PCOS pathophysiology by regulating ovarian innervation, at least partially mediated through the SON.

## Introduction

Due to the limitation of early experimental methods and techniques, it is very difficult to determine the ovarian innervation network, distribution characteristics, and the relationship with follicles and blood vessels during both the developmental process and pathological condition. The emerging technology of three-dimensional tissue transparency and visualization by immunostaining may provide a path to solve the above problems.

Polycystic ovary syndrome (PCOS) is a typical anovulatory disorder in women of reproductive age. The etiology is complex. There is evidence for both epigenetic and environmental factors such as diet and lifestyle, which have shown to play a role in the pathology of the disease. PCOS is frequently associated with metabolic disorders, including obesity, abdominal adiposity, insulin resistance, alongside cardiovascular risk factors (Escobar-Morreale, 2018). Many of the common features of PCOS such as hyperinsulinemia and central obesity are associated with chronic sympathetic nerve hyperactivity. Given the evidence linking sympathetic activation with metabolic disturbances, it is reasonable to speculate that sympathetic neural activity might be increased in PCOS and that such excitation might play a role in the pathogenesis or progression of the syndrome (Lansdown and Rees, 2012).

As a form of traditional Chinese therapy, acupuncture has a long history of use for the treatment of gynecological disorders. In addition to regulating the hypothalamus-pituitary-ovary axis (Feng et al., 2012), acupuncture also directly affects the peripheral tissues such as the ovary, adrenal glands, and adipose tissue (Wang et al., 2015; Kokosar et al., 2018). In the clinic, acupuncture has been shown to have a positive effect on the development of ovarian follicles and the promotion of ovulation in infertile women (Kuang et al., 2013). Low-frequency electro-acupuncture (EA) at both local and distal acupoints has been shown to promote ovulation and increase the likelihood of successful pregnancy (Smith et al., 2016). In addition, previous animal experiments using dihydrotestosterone (DHT)-treated rats showed that EA improves ovarian blood flow, blood redistribution, and angiogenesis, especially in antral follicles (Ma et al., 2018).

In the present study, we used a novel method to study the 3D ovarian innervation and the roles of nerve fibers in folliculogenesis in both control and DHT-induced PCOS-like rats. Ovaries were made transparent using the CUBIC 3D tissue-clearing method for 1 week, and subsequent immunostaining revealed the ovarian vasculature and innervation. The role of innervation by acupuncture was further evaluated by performing unilateral sectioning of the superior ovarian nerve (SON).

## Results

### Ovarian Innervation Increased With Age and Gonadotropin Stimulation, Starting From the Hilum and Extending to Individual Follicles

**FIGURE 1:**
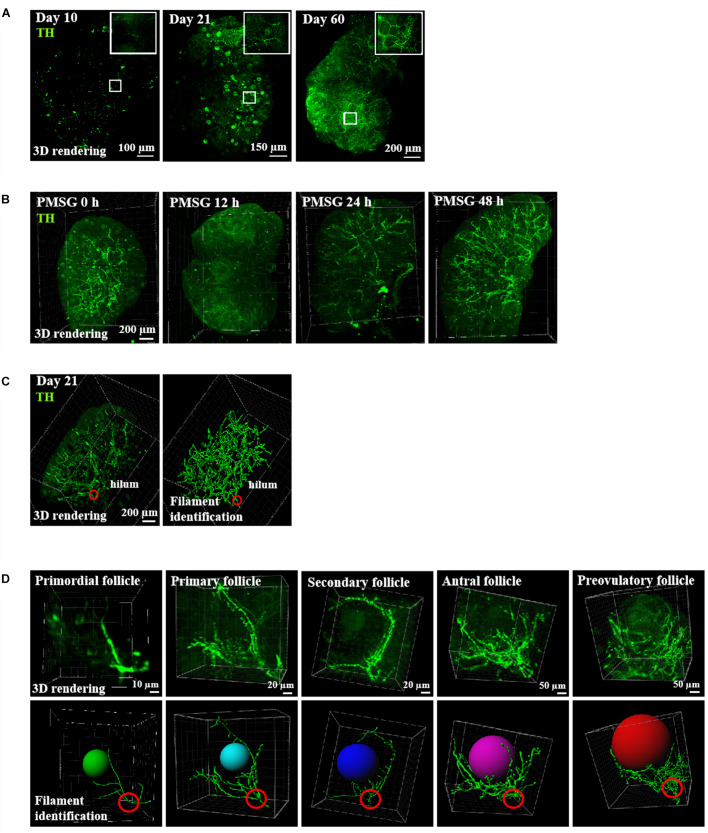
Ovarian innervation of mice at different ages. **(A)** Ovaries from mice at 10, 21, and 60 days of age were cleared using the CUBIC technique prior to immunostaining using TH antibodies. **(B)** Ovarian innervation at different time-points after gonadotropin stimulation. **(C)** Tracing of ovarian nerve fibers of 21-day-old mice. Left panel: TH staining of the whole ovary. Right panel: tracing using the Filament tool in Imaris. **(D)** Tracing of individual follicles from primordial to primary, secondary, antral, and preovulatory stages.

Using mice at different ages, we performed staining of ovarian innervation fibers using tyrosine hydroxylase (TH) antibodies and staining of vascular endothelial cells using CD31 (platelet endothelial cell adhesion molecule-1) antibodies. As shown in Figure 1, strong neuronal fiber staining was seen in the ovarian hilum, in close association with the blood vessels (Figure 1A). During development, both neuronal and vascular staining increased with age. Moreover, in order to investigate the ovarian innervation under the regulation of gonadotropins, we further treated immature mice with pregnant mare serum gonadotropin (PMSG) for 48 h to induce the growth of antral and preovulatory follicles. As shown in Figure 1B, ovarian innervation increased following PMSG treatment with no further increases after human chorionic gonadotropin treatment (data not shown). When combined with tracing of individual follicles using filament identification (Figure 1C), we found that neuronal fibers branched from the hilum and extended to individual follicles throughout the ovary. At the individual follicle level (Figure 1D), each follicle from the primordial to primary and secondary stages showed innervation by a single neuronal fiber.

### EA Restored PCOS Pathophysiology by Promoting the Innervation of Blood Vessels Near the Hilum in PCOS-Like Ovaries

**FIGURE 2:**
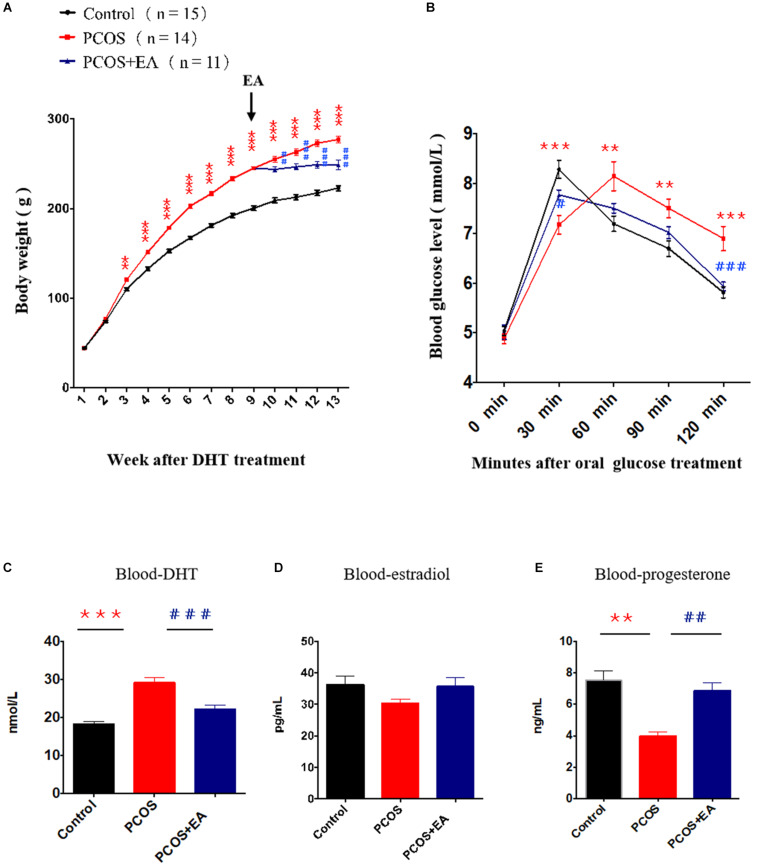
DHT induced a PCOS-like phenotype, and the effect of EA treatment on body weight, insulin resistance and steroids. Rats at 21 days of age were implanted with DHT to induce a PCOS-like phenotype. After 8 weeks of DHT treatment, rats of PCOS + EA received EA treatments for 4 weeks. **(A)** Body weights of rats in each group. Arrow indicated the beginning of the EA trentment. **(B)** The oral glucose tolerance test at the end of the experiment. **(C–E)** Serum levels of DHT, estradiol, and progesterone. **p < 0.01, ***p < 0.001 *vs.* Control; ^##^*p* < 0.01, ^###^*p* < 0.001 *vs.* PCOS.

We implanted capsules containing DHT (15 mg/rat) in rats at 21 days of age. In addition to monitoring body weight changes, we performed an oral glucose tolerance test 12 weeks later. The body weight increased in DHT-treated rats (Figure 2A), and an increase in insulin resistance was also seen (Figure 2B). We also performed EA in DHT-implanted rats (5 days/week from week 8 to week 12 after DHT implantation), and EA rescued the DHT-induced abnormal serum hormone levels in these animals (Figures 2B–E). These results suggest that EA restored DHT-induced metabolic abnormalities in PCOS-like rats.

### DHT Implantation Decreased the Number of Antral Follicles, Pre-ovulatory Follicles, and Corpora Lutea, and This Could Be Rescued by EA

**FIGURE 3:**
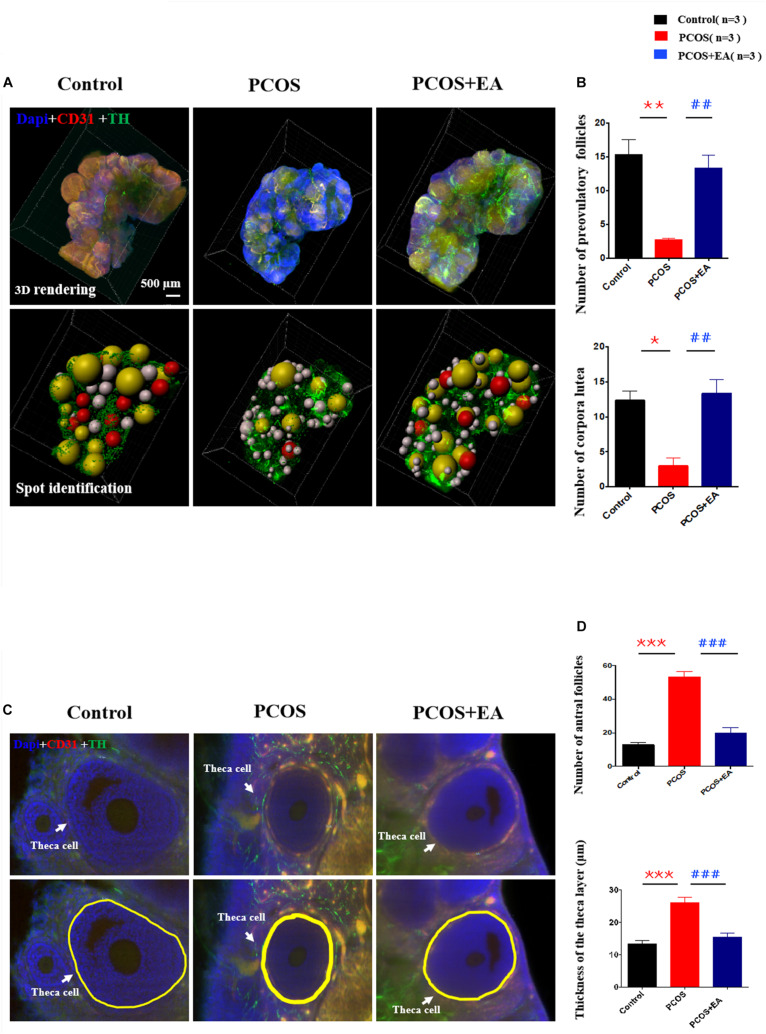
DHT-induced PCOS-like rats showed decreased the number of mature follicles and increased the thickness of the theca cell layer, and the effect of EA treatment. **(A)** Follicles were stained using DAPI (blue) together with antibodies against CD31 (red) or TH (green) for the identification of ovarian innervation and vasculature, respectively. **(B)** The numbers of preovulatory follicles and corpora lutea in the three groups. **(C)** Theca cell layers were increased by DHT and reversed by EA. The thickness of the theca cell layer was measured in 3D images using Imaris (upper panels) from randomly selected antral follicles (*n* = 10). **(D)** The number of antral follicles and the thickness of the theca cell layer around antral follicles were measured in the three groups. *p < 0.05, **p < 0.01, ***p < 0.001 *vs.* Control; ^##^*p* < 0.01, ^###^*p* < 0.001 *vs.* PCOS.

We measured the numbers of mature follicles and corpora lutea in control and DHT-induced PCOS-like rats with or without EA. As shown in Figure 3A and Supplementary Video S1, CUBIC clearing of tissues followed by staining using 4’,6-diamidino-2-phenylindole (DAPI) together with antibodies against CD31 and TH provided a detailed view of the ovarian vasculature and innervation. As shown in Figures 3B,D, DHT implantation increased the number of antral follicles and decreased the numbers of preovulatory follicles and corpora lutea, and EA treatment partially rescued the effects of DHT. To confirm the PCOS-like phenotypes of DHT-induced rats, we measured the thickness of the theca cell layers surrounding the antral follicles. As shown in Figure 3C, there was a ∼twofold increase in the theca cell thickness in DHT-induced rats, and this increase was suppressed by EA.

### DHT Implantation Increased Innervation in the Ovarian Stroma but Decreased Innervation in the Surrounding Follicles, and This Was Rescued by EA

**FIGURE 4:**
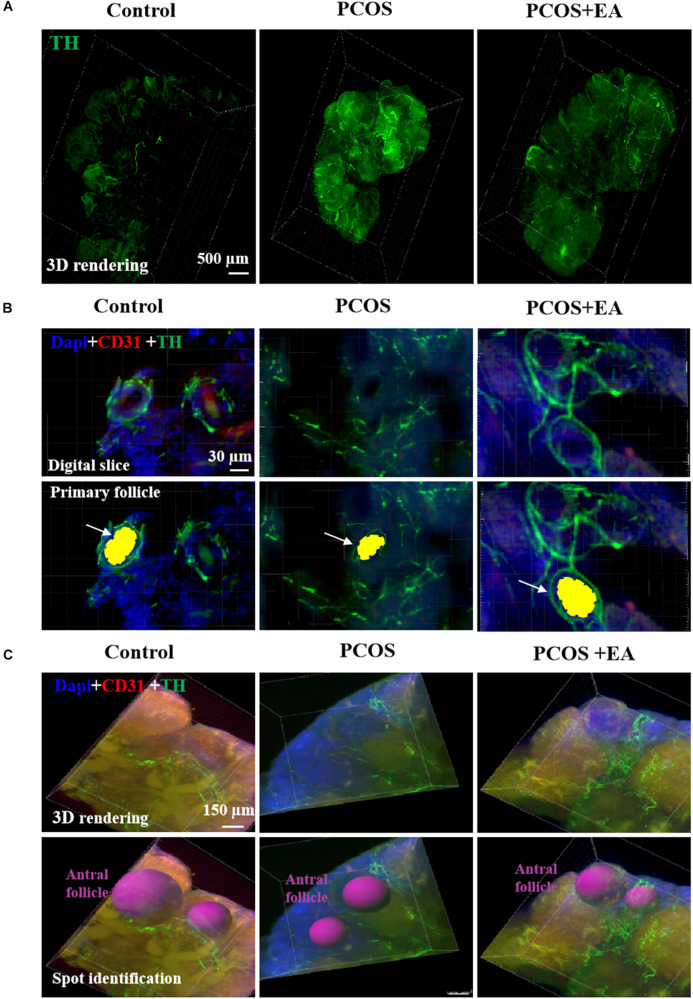
PCOS ovaries showed increased innervation in the ovarian stoma and decreased nerve fibers surrounding the antral follicles, and this was reversed following EA. Ovaries from the three groups were obtained for innervation and blood vessel staining using TH and CD31, respectively. **(A)** Ovarion innervation of the three groups. **(B)** Ovarian innervation of primary follicles. Individual follicles in the three groups were stained using DAPI (blue) together with antibodies against CD31 (red) and TH (green) for identification of blood vessels and neurons, respectively, using the Imaris software. The primary follicle is shown in yellow indicated by arrow. **(C)** Distribution of the neurovasculature surrounding antral follicles, which are marked in red.

Using TH staining, we identified the neuronal fibers in the ovaries of DHT-induced PCOS-like rats with or without EA. As shown in Figure 4A and Supplementary Figures S2A,B, overall TH staining increased after DHT implantation, and EA treatment reversed this effect. As shown in Figure 4B, the increased TH staining was mainly distributed in the stroma but not the surrounding follicles. In antral follicles (Figure 4C), there was a decreased distribution of neurovasculature surrounding individual antral follicles in DHT-induced PCOS-like rats, whereas EA treatment increased the innervation of blood vessels surrounding mature follicles.

### Ovaries in DHT-Implanted Rats Showed Decreased Innervation of the Vasculature Near the Hilum, and This Was Rescued by EA

**FIGURE 5:**
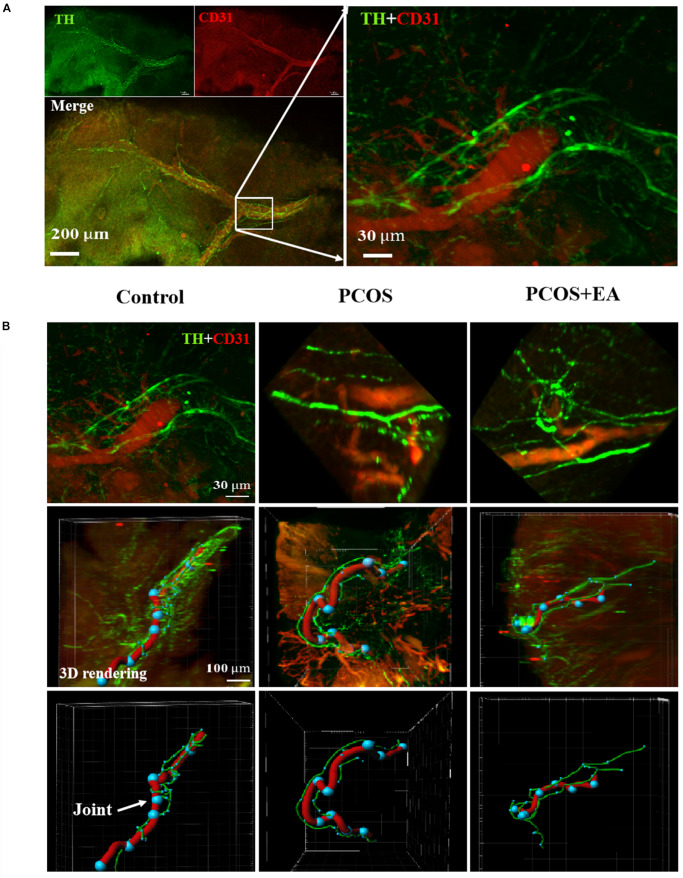
Less joints of innervation of the vasculature near the hilum and follicles in PCOS ovaries, and this is reversed following EA. Ovaries from the three groups were obtained for innervation and blood vessel staining using TH and CD31, respectively. **(A)** The neurovasculature near the hilum of the ovary was visualized with antibodies against CD31 (red) or TH (green). **(B)** The upper panels show the different neurovascular alignments with the hilum in the Control, PCOS, and PCOA + EA groups. The major blood vessel passing through the hilum was identified using the Imaris software (the middle panels), and the joints between vessels and nerve fibers were marked as blue dots indicated by arrow (the bottom panels).

As shown in Figure 5A, neurons in the ovaries were stained using antibodies against TH, and blood vessels were stained using antibodies against CD31. Tracing of neuronal fibers in the ovarian hilum, together with blood vessel staining showed a decreased innervation in the ovaries of DHT-induced PCOS-like rats while EA treatment reversed this decrease (Figure 5B and Supplementary Figure S2C). The major blood vessel passing through the hilum was identified using the Imaris software, and the joints between vessels and nerve fibers were marked as dots.

### The Effect of EA on Increasing Follicle Numbers in PCOS-Like Rats Was Abolished After Sectioning of the SON

**FIGURE 6:**
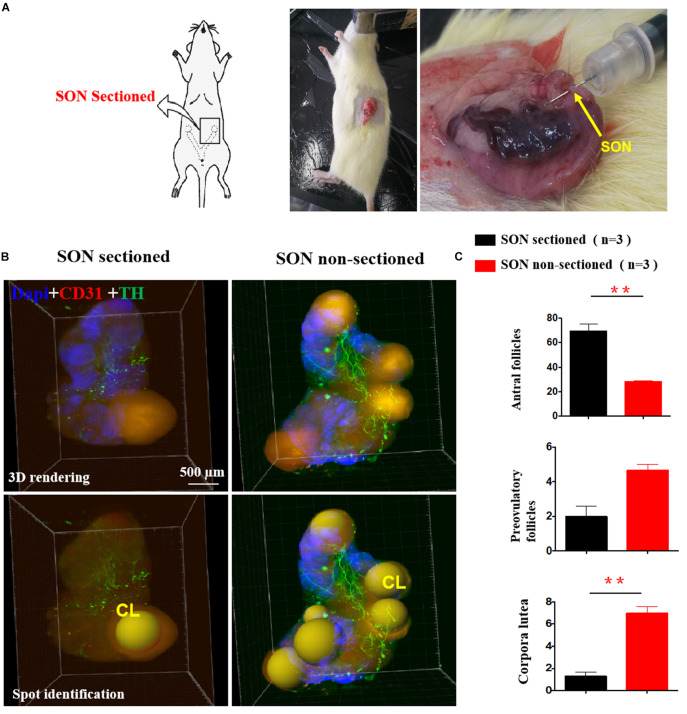
The effect of unilateral SON neurectomy on EA-induced changes in ovarian innervation and vasculature in PCOS-like rats. **(A)** Unilateral SON neurectomy in PCOS + EA rats. Arrow indicated the SON. **(B)** Mature follicles and corpora lutea of the ovaried without SON were decreased compared with those of ovaries with SON. **(C)** Quantitative and statistics analysis of antral, preovulatory follicles, and corpora lutea. **p < 0.01 *vs.* SON sectioned ovaries.

We performed unilateral sectioning of the SON in PCOS + EA rats (Figure 6A), and this was followed by 3D imaging using a lightsheet microscope and analysis using Imaris software to monitor the number of mature follicles. As shown in Figures 6B,C, there was a decrease in the number of antral/preovulatory follicles. Within the corpora lutea, there was also a decrease in SON-sectioned ovaries compared with the contralateral ovaries with the SON non-sectioned. These findings suggest that the SON plays a critical role in mediating the effects of EA.

## Discussion

In the present study, we observed that ovarian innervation increased with age, and the neuronal branching started from the ovarian hilum and extended to individual follicles at different stages. At the individual follicle level, each follicle was primarily innervated by one neuronal fiber. Compared with control rats, ovaries from DHT-induced PCOS-like rats had more cysts follicles (Supplementary Figure S1), fewer preovulatory follicles and corpora lutea. Furthermore, PCOS ovaries showed decreased innervation of blood vessels near the hilum surrounding individual antral follicles, and 80% of the antral follicles had less innervation surrounding the ovarian theca layer, but showed an over-expression of innervation in the ovarian interstitium. In contrast with the PCOS group, EA in PCOS-like rats increased the innervation of blood vessels near the hilum along with the adjacent surrounding theca layers. Sixty antral follicles from three ovaries were randomly selected and observed, and about 53% showed increased localization of innervation surrounding the theca layers. To elucidate the role of ovarian innervation of acupuncture, we further performed unilateral sectioning of the SON in PCOS + EA rats and found that the SON sectioned ovaries had fewer preovulatory follicles and corpora lutea, compared with the SON non-sectioned ovaries suggesting that the integrity of the ovarian nerve is the key to the effects of acupuncture.

Ovarian nerve fibers originate from the neural crest during embryogenesis (Dees et al., 2006), and enter the ovary through the SON to join the ovarian plexus and then, extends to the follicular theca, interstitial glands, and vascular structures (Burden et al., 1983). The extrinsic innervation of the mammalian ovary includes sympathetic, parasympathetic, and sensory fibers (Burden et al., 1983; Ojeda et al., 1985; Lara et al., 1990), while the intrinsic innervation consists of ganglionic polygonal neurons (Dees et al., 1995; D’Albora and Barcia, 1996; D’Albora et al., 2000, 2002; Anesetti et al., 2001). The sympathetic nerves of the mammalian ovary regulate ovarian functions such as follicular maturation, steroid secretion, and ovulation (Lara and Belmar, 1991). Various methods have been developed for evaluating ovarian innervation in both human and animal ovaries. Silver impregnation methods have been used to visualize the ovarian ganglion in mice and rabbits (Dahl and Flaskamp, 1937; Burden, 1978), and in the cat ovary hilum, a vegetative microganglion was described on the basis of the findings obtained with the Nissl method (Hagi-Paraschiv et al., 1959). In the ovaries of adult primates, some intrinsic neurons have tested positive for TH, the rate-limiting enzyme in catecholamine synthesis (Dees et al., 1995), and similar neurons are also present in the human ovary (Anesetti et al., 2001). The number of TH-positive neurons significantly increases along with the development of reproductive capacity at puberty in rhesus monkeys (Dees et al., 2006). Intrinsic neurons of rat ovary first appear in the cortex, and then increase significantly during per-pubertal development and reach their maximum numbers prior to puberty (D’Albora and Barcia, 1996; D’Albora et al., 2000). The expression of a neuron-specific nuclear protein was identified at all postnatal intervals in the rat ovary, 20% are catecholaminergic, while the rest show neuropeptide Y immunoreactivity (D’Albora et al., 2000, 2002). Overall, the results detected neurons-positive or ganglia only in two dimensions by immunohistochemistry. It is difficult to show the global ovarian innervation network and its spatiotemporal relationship with dynamic follicles. The intact ovary transparent and 3D visulazation may provide a new approach in studying the important role of ovarian innervation.

In our previous studies, we used the CLARITY method to visualize follicles and ovarian vascularity (Feng et al., 2017; Ma et al., 2018). However, the passive CLARITY clearing for mouse ovary usually takes about 6–8 weeks for complete tissue clearing and staining. In the present study, we took advantage of the CUBIC method that only takes 1 week to perform, that results in rather rigid ovaries that retain their original structure better than with the CLARITY method. The relatively lower costs of the CUBIC method were also a consideration.

We studied the sympathetic innervation of the ovary in rodents, and the role of ovarian innervation in physiological and pathological variations. Mice at 10, 21, and 60 days of age were used to investigate changes in ovarian follicle development. Ovaries in day 10 mice contained follicles at the primordial, primary, and secondary stages. At day 21, early antral follicles emerged. At day 60, mice reached sexual maturity and had follicles at all stages including mature antral and preovulatory follicles together with corpora lutea. We found that ovarian innervation increased with age, and the neuronal branching started from the ovarian hilum and extended to individual follicles at different stages. For the individual follicle, each follicle was primarily innervated by one neuronal fiber. In addition, even in the course of 48 h of superovulation, the growth speed of a nerve is directly proportionate to the development of a mature follicle and the nerve endings are mostly concentrated on the surface of developing follicle. This suggests that follicular development and ovulation need, not only an abundant blood supply, nutrition, and hormone supply, but also, an intrinsic nerve, which may play a role in stimulating the sensitivity of follicular development initiation (Feng et al., 2017, 2018) and guiding arterioles to the follicular membrane (Eichmann et al., 2005; Mukouyama, 2005).

Polycystic ovary syndrome is a typical disorder of follicular development disease with unknown etiology. DHT-induced PCOS-like rats is a classic animal model which presents reproductive, neuroendocrinologic, and metabolic phenotype (Louise et al., 2007; Feng et al., 2009). In clinic, PCOS patients were reported to be with hyperactivity in the sympathetic nerve system and had decreased activity in parasympathetic and autonomic nerve (Yildirir et al., 2006; Giallauria et al., 2008; Sverrisdottir et al., 2008; Tekin et al., 2008; Dissen et al., 2009). Some clinical symptoms of PCOS such as hyperandrogen, high blood pressure (Sidra et al., 2019), and anxiety (Cinar et al., 2011) are highly related to the sympathetic nerve system or imbalance of sympathetic and the parasympathetic nerve. It has been reported that the density of sympathetic nerve fibers increases in the cystic ovaries of women with PCOS as well as in postmenopausal women (Heider et al., 2001). The increased sympathetic innervation in PCOS is evidenced by increased density of catecholaminergic nerve fibers, altered catecholamine metabolism and/or uptake, and increased nerve growth factor expression (Garcia-Rudaz et al., 1998; Heider et al., 2001; Greiner et al., 2005). Furthermore, it has been shown that ovulation can be induced following an iatrogenic injury, such as the resection (Krishna et al., 2001) or laparascopic laser cauterization of the ovarian medulla fragment that contains nerves innervating the ovary (Donesky and Adashi, 1995). This was effective in PCOS patients in whom cannot respond to hormonal therapy. Although in the development stage, the increase of a nerve is beneficial to follicle activation and development. However, we observed that in PCOS rat ovaries, the accumulation of abnormally increased nerve fibers was concentrated in the stroma of the ovaries, which could not have had a direct effect on the follicles. This may be one of the reasons for the stagnation of PCOS follicle development and the formation of cysts.

In China, acupuncture has a long history in the treatment of gynecological diseases. Since the 1980s, acupuncture has been widely used in China, Europe, and the United States (Wu et al., 2017). A large number of acupuncture experiments have shown that acupoints are rich in nerve endings. Acupuncture plays an important role in regulating “Qi and blood” in traditional Chinese Medicine, and the scientific mechanisms of acupuncture have been proven to be through multiple targets and multiple systems. Our previous studies showed that acupuncture can integrate information from the central nervous system through the sympathetic nerve at the same section of the acupoint (Manneras et al., 2009), regulating various neurotransmitters and neurohormones (Manni et al., 2005; Feng et al., 2012), and can inhibit the abnormal release of gonadotropin-releasing hormone in the hypothalamus (Feng et al., 2009). In the rat ovary, it was also found that 1 min of low-frequency EA could increase the ovarian local blood flow without affecting the mean arterial pressure. Once the ovarian sympathetic nerve was cut, the effect of low-frequency EA on accelerating blood flow was inhibited (Stener-Victorin et al., 2004). Our work also indicated that low-frequency EA also improved the ovarian vascularity and angiogenesis of antral follicles in PCOS (Ma et al., 2018). Repeated EA treatments reduced sympathetic hyperactivity, lowered ovarian NGF levels (Stener-Victorin et al., 2000), and suppressed expression of beta 2, alpha 1, and alpha 2 adrenoceptors (Manni et al., 2005). These findings support the hypothesis that increased sympathetic activity contributed to the development and maintenance of PCOS and that the effects of EA may be mediated by modulation NGF expression of sympathetic outflow to the ovaries (Manneras et al., 2009).

Nerves and blood vessels are closely related in central and peripheral organs, often accompanied in structure, sharing nutrient factors and signaling pathways in function. Attention has been paid to the role of neurovascular coupling. For example, peripheral neurogenic VEGF was required for arterial development, and the arrangement of vascular nerves occurred before angiogenesis (Mukouyama, 2005). The vascularization of organs required angiogenesis and a proper vascular network, which was regulated by the nervous system (Carmeliet, 2003). It has been shown that arteriogenesis was immediately preceded by neurovascular alignment, and the branching patterns of arteries following the nerves (Mukouyama et al., 2002). Previous experiments have shown that nerve-derived VEGFA was required for arteriogenesis in the primitive capillary network of murine limb skin. Arteriogenesis was affected when VEGF signaling was disrupted, while neurovascular alignment was apparently unperturbed (Mukouyama, 2005). Unique with other organs in body, periodic ovulation leads to frequent neogenesis and redistribution of ovarian neurovascular, which may exist in the active neurovascular coupling. Our results showed that both the neuronal and vascular signal increased over the course of development. In PCOS-like rats, there were less joints between the blood vessels and nerves in the hilum and surrounding follicle; but after EA treatment, the number of joints between blood vessels and nerves roughly doubled, which promoted to providing better support for follicular development.

Superior ovarian nerve is the important sympathetic innervation of the ovary and plays a predominant role in follicular development, ovulation, and pregnancy and is involved in the pathogenesis of ovarian diseases (Del Campo et al., 2019). There is an evidence that SON denervation results in almost complete disruption of estrous cycle activity in rats (Forneris and Aguado, 2002), and it has been shown that the pathogenesis of PCOS was related to neural influences on the ovaries via the SON, which regulates ovulation under physiological conditions. Furthermore, changes in serum levels of progesterone, testosterone, and estradiol caused by estradiol valerate treatment were controlled by abdominal wall nerve signals and other signals that reach the ovaries through the SON (Morales-Ledesma et al., 2010). In the present study, we used the same rat by only cutting one side of the ovarian SON in order to exclude the impact of hormone fluctuations on ovarian follicles. Both ovaries were intact and could respond to hormones. PCOS ovaries with SON showed more preovulatory follicles and corpora lutea than those of the other PCOS ovary without SON after 4 weeks of EA treatment, which suggests that the effect of EA was based on the integrity of the nervous system.

## Conclusion

Ovarian innervation likely played an important role in folliculogenesis, and EA might restore PCOS pathophysiology by regulating ovarian innervation, at least partially mediated through the SON.

## Materials and Methods

### Animals

Female C57 mice and wistar rats were from Shanghai SLAC Laboratory Animal Co. Ltd. (Shanghai, China). Animals were housed in animal facilities at Fudan University under 12 h dark/light with free access to food and water. To investigate gonadotropin regulation of folliculogenesis, 21-day-old mice were injected with 5 IU of eCG (HZDWYC, Hangzhou, China, Lot: 080231281) into the caudal vein. The ovaries were harvested from mice at 0, 12, 24, and 48 h. After another 48 h, we injected the mice with 10 IU hCG (SANMA, Harbin, China, Lot: 181111), and the ovaries were taken at 6 and 12 h after hCG injection (Hsueh et al., 1994). To investigate the different stages of mouse folliculogenesis, we obtained 10, 21, and 60-day-old mouse ovaries. The ovaries were further processed using CUBIC and immunostaining to visualize the ovarian nerves. To evaluate the effect of ovulation induction, we counted the number of follicles at different stages.

The use of animals and the experimental design were approved by the Animal Ethics Committee, School of Basic Medical Sciences of Fudan University, China (ID: 20150119-019, approval date: 19 January 2015).

### Establishment of PCOS-Like Rats

Female Wistar rats (aged 21 days, Shanghai SLAC Laboratory Animal Co. Ltd., Shanghai, China) were housed under controlled conditions (21–22°C, 55–65% humidity, 12 h light/12 h dark, and free access to food and water). All rats were randomly divided into three groups: Control (*n* = 15), PCOS (*n* = 14), and PCOS + EA (*n* = 11). Based on earlier experiments (Louise et al., 2007), silicone tubes containing DHT (15 mg, slow-releasing for 75 days, 1 cm in length, 2 mm in diameter) were implanted subcutaneously into the necks of rats to establish a PCOS-like phenotype.

Rats were anesthetized with isoflurane, the flabby skin of the neck was incised, the tube was imbedded, and the wound was sutured. Body weight and metabolic indexes were measured (Figure 2B and Supplementary Table S3).

### Oral Glucose Tolerance Test (Cui et al., 2018)

After fasting overnight (10–12 h), glucose levels in the tail vein blood were measured using a blood glucose meter (ACCU-CHECK Performa, Roche). One researcher securely held the animal and cleaned the tail, and another researcher prepared the blood glucose meter and took the measurement. Basal blood glucose levels were measured prior to administration of 50% oral D-glucose (2 g/kg body weight), and measurements were taken at 30, 60, 90, and 120 min after glucose administration. After each measurement, the tail was covered with gauze and kept at room temperature.

### Unilateral SON Denervation (SONx)

This experiment was carried out at 8 weeks after DHT pellet implantation before acupuncture. Some animals in the PCOS + EA group were randomly assigned into PCOS + EA unilateral SON transection group. An individual animal was lying with the left ovary exposed following a horizontal incision of about 2 cm and removal of abdominal fat. Based on the location of the SON nerve innervating the ovary (Uchida and Kagitani, 2014; Pastelin et al., 2017) and as shown in Figure 6A, the SON was cut with scissors. This was following by suturing the wound and kept warm to avoid infection.

### Low-Frequency EA (Feng et al., 2009)

Rats in the PCOS + EA group were treated with low-frequency EA from Monday to Friday for 4 weeks (8–11 a.m., for a total of 20 treatments). Under isoflurane anesthesia, rats had single-use sterile acupuncture needles inserted into the bilateral acupoints “Guilai” (ST 29) and “Sanyinjiao” (SP 6). Home-made restraints were used to hold the rats in place and to maintain their posture. Acupuncture needles were inserted to a depth of 0.5–0.8 cm in the posterior part of the medial tibia for SP 6 and in the bilateral part below the umbilical for ST 29. The needles were attached to an electrical stimulator (HANS-LH202, Huawei Co., Ltd., Beijing, China) under 2 Hz/2 mA for 30 min. Rats were concious during the EA treatments.

### CUBIC 3D Imaging

We performed the CUBIC clearing method described by Susaki et al. (2015). Mice or rats were perfused with phosphate buffer saline (1 × PBS, 50 or 200 ml, respectively, at 4°C) containing 10 U/ml of heparin to wash out the blood, followed by 4% paraformaldehyde (PFA) at 4°C. Ovaries were carefully dissected and immersed in 4% PFA at 4°C for 24 h. Before clearing, the tissues were washed with 1 × PBS/0.01% sodium azide for 2–4 h at 37°C with shaking to remove the remaining PFA. The samples were then cleared with diluted reagent 1(CUBIC clearing solution) with shaking at 37°C for 3 h, followed by washing with reagent 1 until the tissue turning clear. The samples were then washed with 1 × PBS with 0.01% sodium azide for 2 h three times. The samples were then incubated with primary rabbit polyclonal antibodies (Supplementary Table S1) for 2 days, washed in 1 × PBS for 1 day, and then incubated with secondary antibodies for 2 days, all with shaking at 37°C (Supplementary Table S2). After immunofluorescence staining, diluted reagent 2 was applied with shaking at 37°C until the samples sank to the bottom. The samples were then transferred to reagent 2 for at least 1 day until turning completely clear. Cleared samples were stored in reagent 2 at 4°C for imaging.

3D images were collected using a Lightsheet Z.1 confocal microscope (Zeiss, Oberko, Germany). For each imaging, an ovary was attached to the bracket with glue, and a 5 × objective was used with a working distance of 3 mm. We set the *Z*-axis of the ovary in the “*Z*-stack” tool, and the “Multiview-Setup” tool was used to set the *X*- and *Y*-axes. To get a better quality image, we set the overlap parameter to 10%. After scanning, the data were saved in CZI format and exported to TIFF format using the Arivis software (Arivis AG, Munich, Germany). Digital images were analyzed and reconstructed using Imaris software (v. 9.0, Bitplane, Zurich, Switzerland). The Imaris Spot algorithm was used to semi-manually determine the identity of the follicle, and the Filament algorithm to reconstruct the ovarian innervation (Feng et al., 2017; Ma et al., 2018).

### Data Analysis and Statistics

Statistical analysis was performed in GraphPad Prism (v 7.0a, GraphPad Software, Inc., San Diego, CA, United States). One-way analysis of variance using the *post hoc* Tukey test was performed to calculate the significance between the groups. All data are expressed as the mean ± standard error of the mean, and *p* < 0.05 is considered statistically significant.

## Data Availability Statement

The raw data supporting the conclusions of this article will be made available by the authors, without undue reservation, to any qualified researcher.

## Ethics Statement

The animal study was reviewed and approved by the Animal Ethics Committee, School of Basic Medical Sciences of Fudan University, China (ID: 20160225-013, approval date: 25 February 2016).

## Author Contributions

YF and XT conceived the experiments, designed the project and protocols, and developed the collaborations. YL, XX, JS, WH, TM, PC, WL, ZP, MX, and FZ performed the experiments. XT and WH analyzed the results. TM and YF wrote the manuscript. XL provided scientific oversight and guidance and edited the manuscript. XT and YF are the guarantors of this work and as such had full access to all of the data in the study and take responsibility for the integrity of the data and the accuracy of the data analysis.

## Conflict of Interest

The authors declare that the research was conducted in the absence of any commercial or financial relationships that could be construed as a potential conflict of interest.

## Supplementary Material

FIGURE S1 CUBIC transparent process and immunostaining control. **(A)** The transparent process of the ovary by CUBIC. **(B)** Negative control with secondary antibody only and non-targeting IgG with DAPI (blue). **(C)** Magnification of cystic follicles of PCOS ovaries.

FIGURE S2 Quantitative and statistics analysis of ovarian innervation. **(A)** Statistics of average length on the ovarian innervation in three groups. **(B)** Statistics of average volume on the ovarian innervation in three groups. **(C)** Statistics of joints between vessels and nerve fibers in three groups. **p* < 0.05, ***p* < 0.01 *vs.* Control; ^#^*p* < 0.05 *vs.* PCOS.

TABLE S1 List of primary antibodies.

TABLE S2 List of secondary antibodies.

TABLE S3 List of metabolic indexes.

VIDEO S1 Ovary stained using DAPI (blue) together with antibodies against CD31 (red) or TH (green) for the identification of ovarian innervation and vasculature, respectively.
