# Supplementary figures and images for: Ovarian Innervation Coupling With Vascularity: The Role of Electro-Acupuncture in Follicular Maturation in a Rat Model of Polycystic Ovary Syndrome
Source: Front Physiol. 2020 May 29;11:474. doi: 10.3389/fphys.2020.00474 (PMC7273926; doi:10.3389/fphys.2020.00474)

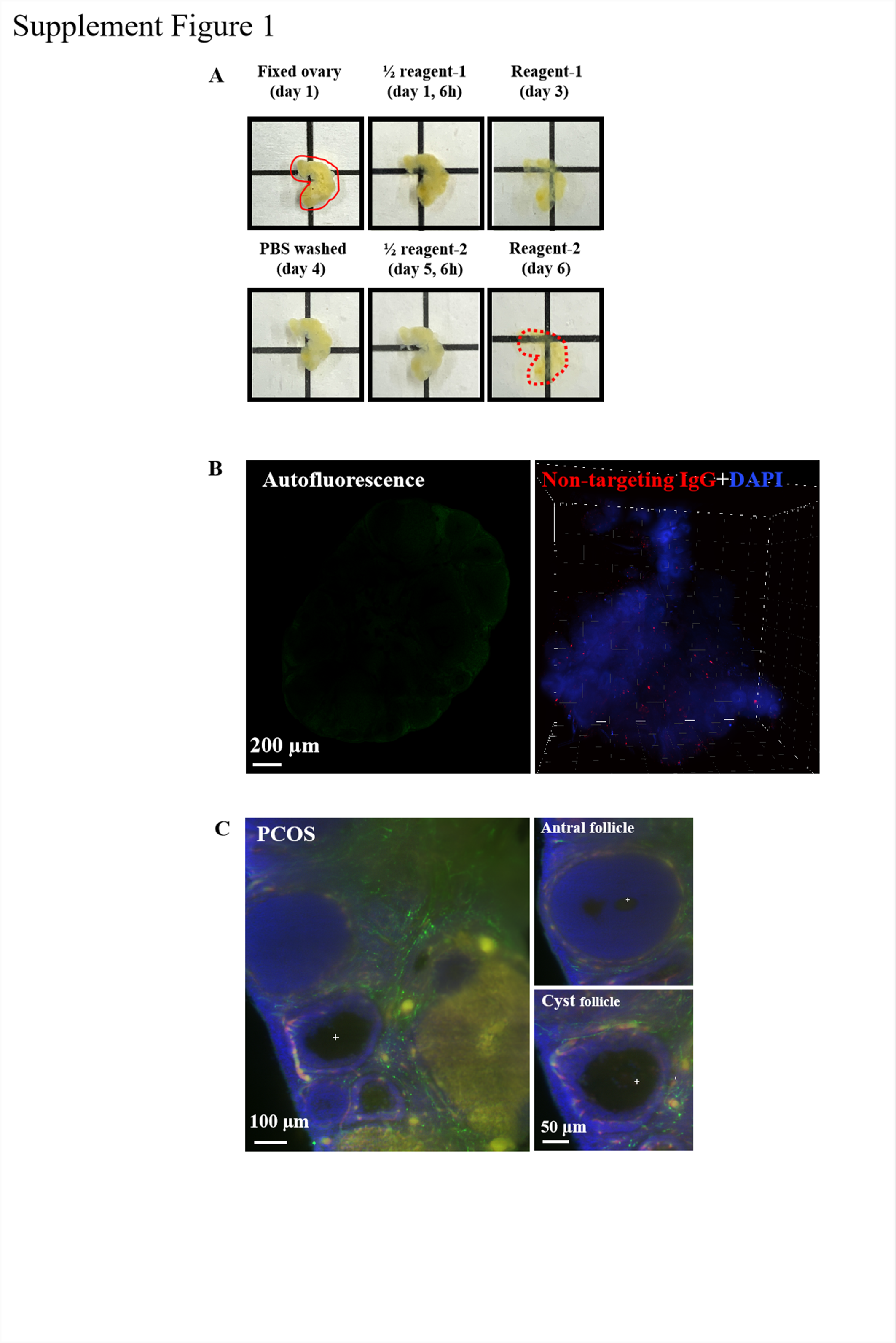

Supplement: FIGURE S1 — CUBIC transparent process and immunostaining control. (A) The transparent process of the ovary by CUBIC. (B) Negative control with secondary antibody only and non-targeting IgG with DAPI (blue). (C) Magnification of cystic follicles of PCOS ovaries. [file Image_1.TIF]

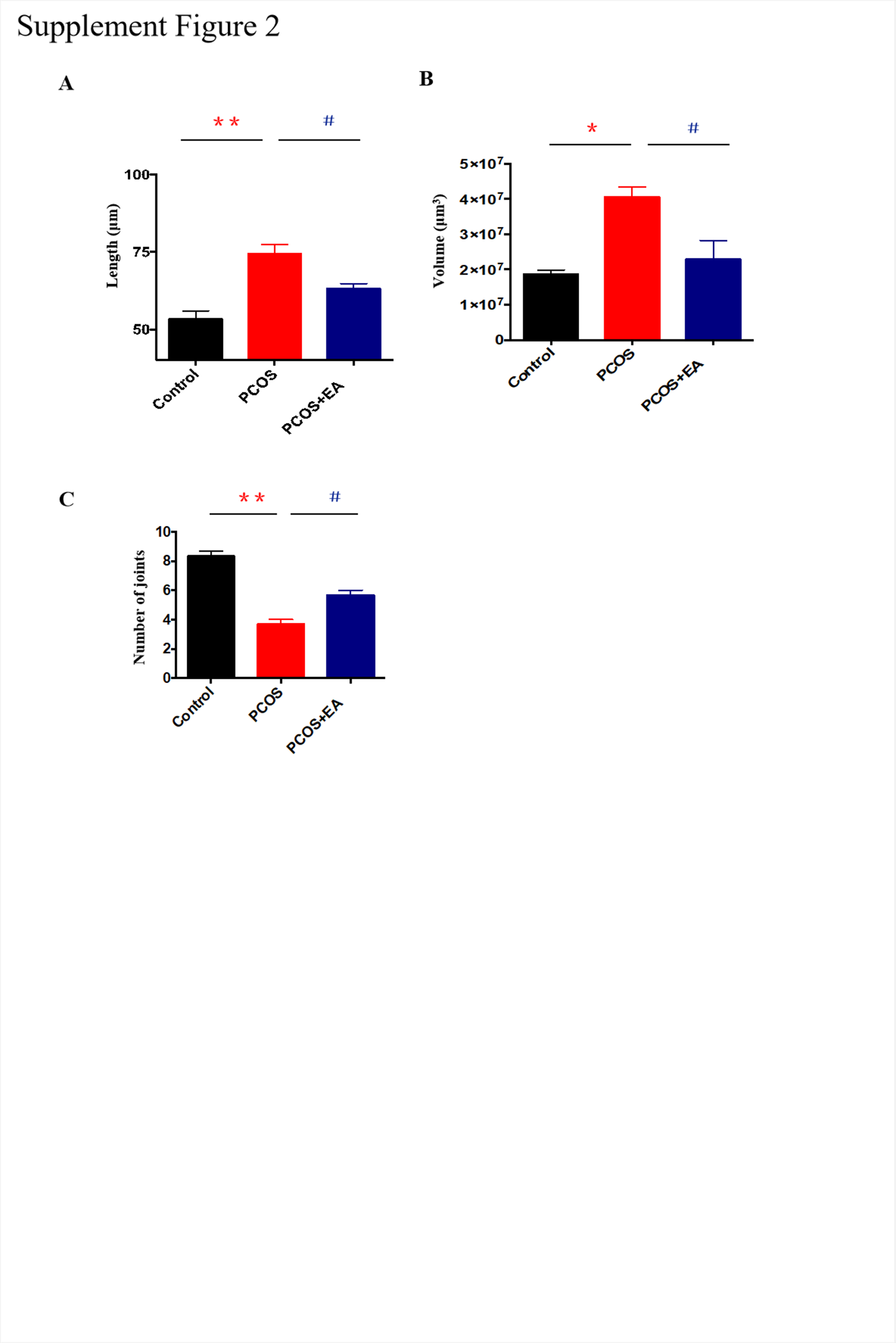

Supplement: FIGURE S2 — Quantitative and statistics analysis of ovarian innervation. (A) Statistics of average length on the ovarian innervation in three groups. (B) Statistics of average volume on the ovarian innervation in three groups. (C) Statistics of joints between vessels and nerve fibers in three groups. *p < 0.05, **p < 0.01 vs. Control; #p < 0.05 vs. PCOS. [file Image_2.TIF]
