# Supplementary material for: Ovarian Innervation Coupling With Vascularity: The Role of Electro-Acupuncture in Follicular Maturation in a Rat Model of Polycystic Ovary Syndrome
Source: Front Physiol. 2020 May 29;11:474. doi: 10.3389/fphys.2020.00474 (PMC7273926; doi:10.3389/fphys.2020.00474)
Supplement: TABLE S1 — List of primary antibodies. [file Data_Sheet_1.PDF]

Supplementary Table 1. List of primary antibodies

Antisera	Code	Host species	Dilution	Company
TH	ab112	Rabbit	1:100	Abcam (LOT:GR3244479-9)
CD31	MAB1398Z	Hamster	1:100	Merck Millipore (LOT:3170789)
Non-targeting IgG	ab6199	Rabbit	1:200	Abcam (LOT:GR201145-7)

Supplementary Table 2. List of secondary antibodies

Antibody	Dilution	Company
Donkey anti-rabbit IgG	1:100	Thermo Fisher Scientific (LOT:1608464)
Goat anti-hamster IgG	1:100	Thermo Fisher Scientific (LOT:2041067)

Supplementary Table 3. List of metabolic indexes

**Variable**	**Control**	**PCOS**	**PCOS+EA**
**Weights**			
Body weight (kg)	0.222 ± 0.003	0.274 ± 0.003***	0.248 ± 0.005###
**Glucose metabolism**			
Fasting glucose (mmol/)	5.038 ± 0.089	4.883 ± 0.094	5.015 ± 0.148
Fasting insulin (uIU/ml)	35.879 ± 1.634	31.873 ± 1.589	32.939 ± 1.932
**Lipid profile**			
TC (mmol/l)	1.441 ± 0.068	1.955 ± 0.130***	1.513 ± 0.050###
TG (mmol/l)	0.688 ± 0.045	2.185 ± 0.148***	1.399 ± 0.211###
HDL (mmol/l)	1.051 ± 0.057	1.028 ± 0.050	0.851 ± 0.027
LDL (mmol/l)	0.148 ± 0.021	0.176 ± 0.042	0.149 ± 0.005
**Liver function**			
ALT (U/L)	56.048 ± 4.022	200.933 ± 5.2558***	65.079 ± 9.489###
AST (U/L)	171.460 ± 2.019	302.545 ± 3.723***	169.748 ± 1.764###

All values are means ± SEM.

***$p<0.001$ *vs.* Control ### $p<0.001$ *vs.* PCOS
